# P-256. Performance of Anthropometric Measures for Screening Excess Visceral Adipose Tissue in Women with HIV

**DOI:** 10.1093/ofid/ofaf695.477

**Published:** 2026-01-11

**Authors:** Jihad Slim, Paul Bellafiore, Bereket K Tewoldemedhin, Vadim Belinschi, James Fallon, Kevin Leyden, Ronald Poblete

**Affiliations:** Saint Michael’s Medical Center, Newark, NJ, USA, Newark, NJ; Saint Michaels Medical Center, Newark, New Jersey; Saint Michaels Medical Center, Newark, New Jersey; Saint Michaels Medical Center, Newark, New Jersey; Saint Michaels Medical Center, Newark, New Jersey; NJCRI, Newark, New Jersey; Northern Jersey Community Research Initiative NJCRI, Newark, New Jersey

## Abstract

**Background:**

The prevalence of Excess Visceral Adipose Tissue (VAT) is increasing in People living with HIV (PWH), however, data supporting cost-effective methods for identifying excess VAT is limited.^1-2^ Excess VAT is associated with multiple comorbidities and metabolic syndrome.^1-3^ Directly measuring VAT through CT scan or DEXA scan can be costly and impractical.^2-3^ A potential solution is to use anthropomorphic measurements to predict excess VAT, as illustrated in the VAMOS Study.^3-4^ In the VAMOS study, the authors found that Waist Circumference (WC) and Waist-to-Hip Ratio (WHR) are the best predictors for excessive VAT in men. However, the study only had 13 women, and WC was the best predictor, but not WHR. Our study aims to expand on the VAMOS cross-sectional study with a data pool focused on women with HIV.

**Figure:**
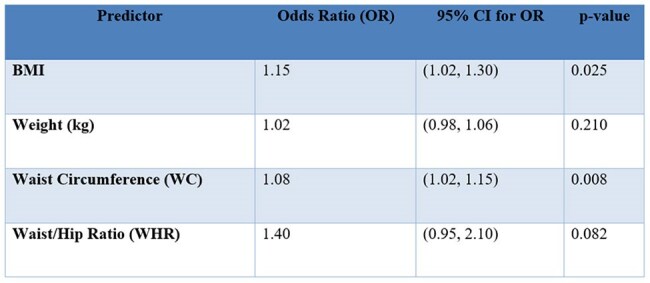
Performance of BMI, Weight, WC and WHR in predicting excess VAT in women with HIV

**Figure:**
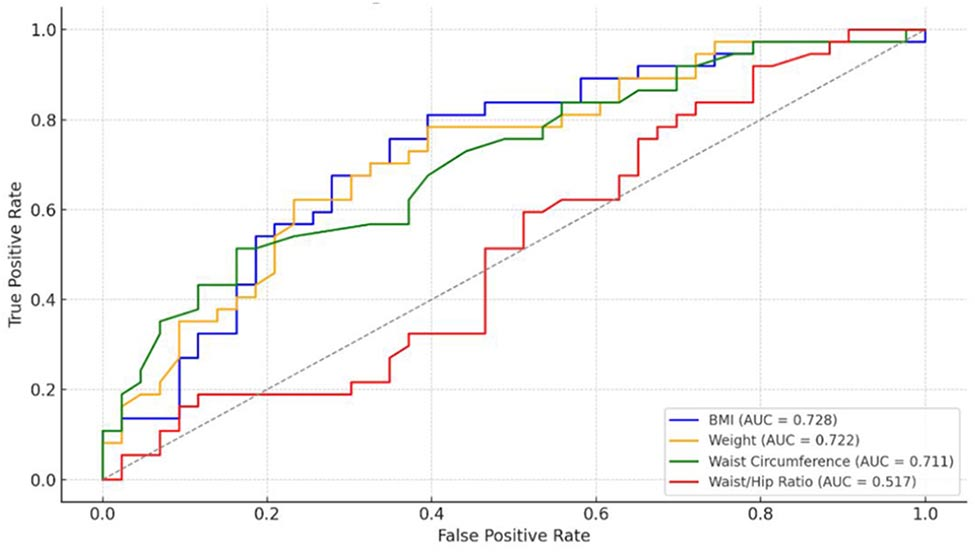
ROC curves evaluating performance of anthropometric measures in predicting excess VAT in women with HIV The AUC was calculated to evaluate BMI, weight, WC and WHR to identify excess VAT in women with HIV

**Methods:**

A multicenter cross-sectional study was conducted in PWH. Eligible participants were over 18 years old and had HIV viral load < 200 c/ml. Individuals with chronic hepatitis B, C or excessive alcohol consumption (AUDIT score >5) were excluded. Excess VAT was quantified by Visceral Adiposity Index (VAI). Weight, BMI, WC, and WHR were calculated for women. Statistical analysis of each variable was done using multivariate analysis, and Receiver Operating Curve (ROC) was done for each anthropometric measurement in the women data set.

**Results:**

A total of 245 participants were enrolled in the study, of which 80 (32.6%) were women, and were included in this analysis.

Median age is 58 years. 67% identify as black and 30% identify as Hispanic.

BMI and WC were statistically significant predictors of excess VAT, while Weight and WHR did not show a statistically significant correlation (Table 1).

Comparing AUC, BMI: 0.728, and WC: 0.711 all perform well; however, WHR: 0.517, viewed alone, is not a strong predictor (Figure 1).

**Conclusion:**

Our study adds evidence that WC and BMI are effective predictors of excess VAT compared to weight and WHR in women living with HIV and further reinforces the findings in the VAMOS study.

**Disclosures:**

Jihad Slim, MD, FACP, gilead: Honoraria|merck: Honoraria|Thera: Honoraria|ViiV: Honoraria Kevin Leyden, RN, RN, Abbvie Inc.: Advisor/Consultant|Gilead Sciences: Advisor/Consultant|Gilead Sciences: Grant/Research Support

